# Validating the agreement between the geriatric trauma frailty index and four published frailty scores in the Chinese geriatric trauma population

**DOI:** 10.1186/s12877-022-02819-9

**Published:** 2022-03-04

**Authors:** Fangjie Zhao, Bihan Tang, Xu Liu, Zhifeng Zhang, Lulu Zhang

**Affiliations:** 1 Naval Medical University Faculty of Health Service, 800 Xiangyin Rd, Shanghai, 200433 China; 2 Shanghai Medical Emergency Center, Shanghai, China

**Keywords:** Frailty scores, Geriatric, Trauma, Elderly population, Agreement

## Abstract

**Background:**

In the geriatric patient population, frailty significantly affects a patient’s prognostic outcome. This study aimed to compare the consistency of our constructed geriatric trauma frailty index with previously published indexes.

**Methods:**

The geriatric trauma frailty index (GTFI) was compared with four previously published frailty indexes, i.e., the hospital frailty risk score (HFRS), Fried index, trauma-specific frailty index (TSFI), and 11-item modified frailty index (mFI) using the Bland-Altman method, intraclass correlation coefficient (ICC), and kappa consistency test. The indexes were calculated based on data collected from 101 questionnaires and medical records from 101 geriatric trauma patients at a tertiary hospital in Shanghai.

**Results:**

Among the 101 geriatric trauma patients, 64 (63.4%) were women, with a mean age of 71.18 (SD = 9.89) years and mean length of stay (LOS) of 7.51 (SD = 3.89) days. The mean scores of GTFI score(≥ 1.3045 as frail), Fried index score(≥3 items as frail), TSFI score(≥ 4 as frail), and mFI (≥ 3 as frail),were 0.86 (SD = 1.51), 0.76 (SD = 1.07), 1.76 (SD = 1.96), and 1.29 (SD = 1.17). respectively. The GTFI score had good consistency with the HFRS (ICC: 0.716, 95% confidence interval [CI]: 0.596, 0.799, kappa: 0.608, 95% CI: 0.449, 0.766), fair consistency with the TSFI (ICC: 0.407, 95% CI: 0.227, 0.562, kappa: 0.460, 95% CI: 0.239, 0.672), and poor consistency with the mFI (ICC: 0.286, 95% CI: 0.097, 0.455, kappa: 0.305, 95% CI: 0.069, 0.525) and Fried index score (ICC: 0.256, 95% CI: 0.063, 0.426, kappa: 0.188, 95% CI: − 0.028, 0.408).

**Conclusions:**

Different frailty indexes are based on different concepts of frailty and cannot be assumed to be interchangeable. There is still no gold standard for the current assessment methods of frailty, but it can be compared based on the understanding in terms of the concepts and measures used in each.

**Supplementary Information:**

The online version contains supplementary material available at 10.1186/s12877-022-02819-9.

## Introduction

Decreased physiologic reserve and frailty in elderly patients reduces their ability to withstand external stressors, making clinical decision-making in these patients challenging [1, 2]. As the Chinese population continues to age, the elderly trauma patient population comprises an increasing proportion of cases in the emergency department [3–6]. Due to the impacts of both frailty and the severity and complexity of trauma injuries, geriatric trauma patients have a certain degree of difference.

Predicting the prognostic outcomes of hospitalized patients is an important part of the daily management of elderly trauma patients that needs to begin immediately after patient admission [7]. Early understanding of these prognostic outcomes can help in communication with family and in mobilizing hospital resources [7]. In addition, a frailty assessment method based on International Statistical Classification of Disease and Related Health Problems, 10th Revision (ICD-10) codes is needed in the development of hospital electronic medical records [8]. In past studies, we developed the geriatric trauma frailty index (GTFI) based on 28,179 records of geriatric trauma in-hospital patients in National Emergency Department Sample NEDS 2016. We validated it using 113,088 cases in NEDS 2016 and 14,827 cases from 11 hospitals in the Shanghai Trauma Emergency Medical Association as a national validation cohort and a local validation cohort, respectively, to determine the frailty of elderly trauma patients and patients who may have poor prognostic outcomes [9]. The results showed that GTFI has a good ability to assess the frailty of geriatric trauma patients, and to predict the length of stay (LOS) (> 14 days) and in-hospital mortality of Chinese geriatric trauma patients. The GTFI evaluation table is shown in the appendix (Appendix 5).

This study aimed to validate the consistency of the GTFI with existing major frailty indexes, including the hospital frailty risk score (HFRS) [8], which is also based on ICD-10 codes, Fried index (based on the frailty phenotype) [10], trauma-specific frailty index (TSFI) [7], and 11-item modified frailty index (mFI) [11].

## Methods

### Study design

After developing the GTFI [9], the research team conducted an investigation at a tertiary hospital in Shanghai from November 2019 to January 2020. Included patients came from two departments, the emergency department and trauma orthopedics department. Medical records were analyzed for patients who met the following inclusion criteria: 1) the main diagnostic ICD-10 codes include S00-S99 (excluding S00, S10, S20, S30, S40, S50, S60, S70, S80, and S90), T07, T14, T20-T28, T30-T32, and T79.A1-T79.A9, namely trauma patients (according to the definition of trauma in the National Trauma Database) [12]; 2) hospitalized patients; 3) geriatric patients (age ≥ 65 years in NEDS and ≥ 60 years in Shanghai Trauma Emergency Medical Association STEMA). We collected 102 questionnaires and medical records, and excluded 1 questionnaire due to incomplete information. In the end, valid questionnaires and medical records were obtained from 101 patients (99.02%).

### Data collection

First, we conducted a questionnaire survey, which was formed based on a review of the literature including the Fried index [5], TSFI Trauma Specific Vulnerability Scale [13], and mFI [11] and calculated the corresponding scores to evaluate the frailty of patients.

Second, we collected relevant information from the patients’ medical records to evaluate their GTFI scores. The data included: gender, age, LOS, total cost, ICD-10 diagnostic codes, and codes for external causes of injury and poisoning. We also calculated their HFRS [8] and Charlson comorbidity score [13].

We then conducted descriptive analysis on the patients’ demographic information and their frailty-related indexes. We also verified the consistency of the GTFI with other existing major indexes (Fried index, TSFI, mFI, and HFRS) (Appendices 1, 2, 3, 4 and 5).

### Statistical analyses

The questionnaires and medical records were uniformly coded after being collected, the data were entered using EpiData by two persons, and any inconsistent entries were checked. The Bland-Altman method comparison, intraclass correlation coefficient (ICC), and kappa consistency test were used. Statistical analyses were performed using SPSS 21.0 (SPSS Inc., Chicago, IL, USA), and *p* < 0.05 was considered statistically significant.

## Results

Among the 101 geriatric trauma patients, 64 (63.4%) were women, with a mean age of 71.18 (SD = 9.89) years, mean LOS of 7.51 (SD = 3.89) days, and mean total cost of 59,442.08 (SD = 40,958.43). For the frailty-related indexes, the average GTFI score was 0.86 (SD = 1.51) (total score 30.306), the average Fried index score was 0.76 (SD = 1.07) (total score 5), the average TSFI score was 1.76 (SD = 1.96) (total score 15), and the average 11-item modified frailty index score was 1.29 (SD = 1.17) (total score 11) (Table 1).

**Table 1:** Demographic characteristics and frailty status of 101 geriatric trauma patients

Categories		Number (%)
Total		101
Gender	Male	37 (36.6)
	Female	64 (63.4)
Age	60–74	65 (64.4)
	75–89	32 (29.7)
	> 90	4 (3.9)
	Mean (SD)	71.18 (9.89)
Length of stay (LOS)	0–7 days	53 (52.5)
	8–14 days	45 (44.5)
	> 14 days	3 (3.0)
	Mean (SD)	7.51 (3.89)
Total cost (¥)	0–30,000	29 (28.7)
	30,000–60,000	8 (7.9)
	60,000–90,000	44 (43.6)
	> 90,000	20 (19.8)
	Mean (SD)	59,442.08 (40,958.43)
Charlson comorbidity index (SD)		3.00 (1.36)
Geriatric Trauma Frailty Index (GTFI)	Non-frail (< 1.3045)	82 (81.2)
	Frail (> 1.3045)	19 (18.8)
	Mean (SD)	0.86 (1.51)
Hospital Frailty Risk Score (HFRS)	Non-frail (< 5)	66 (65.4)
	Frail (> 5)	35 (34.7)
	Mean (SD)	4.04 (2.34)
Fried Index (FI)	Non-frail (< 3)	88 (87.1)
	Frail (> 3)	13 (12.9)
	Mean (SD)	0.76 (1.07)
Trauma-Specific Frailty Index (TSFI)	Non-frail (< 4)	84 (83.2)
	Frail (> 4)	17 (16.8)
	Mean (SD)	1.76 (1.96)
11-Item Modified Frailty Index (mFI)	Non-frail (< 3)	83 (82.2)
	Frail (> 3)	18 (17.8)
	Mean (SD)	1.29 (1.17)

**Fig. 1:**
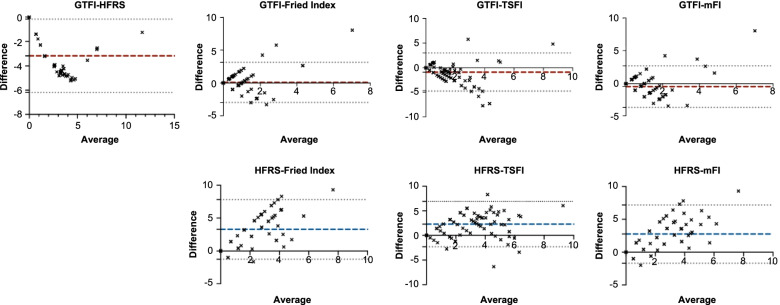
Bland-Altman method comparisons between GTFI, HFRS and other frailty indexes

We evaluated the consistency between the different indexes using the Bland-Altman method comparison (results are shown in Fig. 1). The GTFI score had a good consistency with the HFRS (ICC: 0.716, 95% confidence interval [CI]: 0.596, 0.799), general consistency with the TSFI score (ICC: 0.407, 95% CI: 0.227, 0.562), and poor consistency with both the mFI (ICC: 0.286, 95% CI: 0.097, 0.455) and Fried index (ICC: 0.256, 95% CI: 0.063, 0.426) score. These correlation values are better than those of HFRS. (Table 2).

**Table 2:** The consistency between GTFI, HRFS and other frailty indexes (scores)

Index	Compared index	Intraclass Correlation Coefficient (ICC) (95%CI)	*P* value
Geriatric Trauma Frailty Index (GTFI)	Hospital Frailty Risk Score (HFRS)	0.716 (0.596, 0.799)	< 0.001
	Fried Index (FI)	0.256 (0.063, 0.426)	0.002
	Trauma-Specific Frailty Index (TSFI)	0.407 (0.227, 0.562)	< 0.001
	11-Item Modified Frailty Index (mFI)	0.286 (0.097, 0.455)	0.002
Hospital Frailty Risk Score (HFRS)	Fried Index (FI)	0.195 (0, 0.375)	0.025
	Trauma-Specific Frailty Index (TSFI)	0.404 (0.227, 0.555)	< 0.001
	11-Item Modified Frailty Index (mFI)	0.254 (0.063, 0.428)	0.005

In terms of the consistency of frailty status between different indexes, the GTFI score (≥ 1.3045 as frail) had a strong consistency with the HFRS (≥ 5 as frail) (kappa 0.608, 95% CI 0.449, 0.766), moderate consistency with the TSFI (≥ 4 as frail) score, (kappa 0.460, 95% CI 0.239, 0.672), general consistency with the 11-item mFI (≥ 3 as frail), (kappa 0.305, 95% CI 0.069, 0.525), and poor consistency with the Fried index (≥3 items as frail), (kappa 0.188, 95% CI -0.028, 0.408) score. These were better than that of HFRS. (Table 3).

**Table 3:** The consistency between GTFI, HRFS and other frailty indexes (frailty status)

Index	Compared index	Kappa value	95%CI
Geriatric Trauma Frailty Index (GTFI> 1.3045)	Hospital Frailty Risk Score (HFRS> 5)	0.608	(0.449, 0.766)
	Fried Index (FI > 3)	0.188	(−0.028, 0.408)
	Trauma-Specific Frailty Index (TSFI> 4)	0.460	(0.239, 0.672)
	11-Item Modified Frailty Index (mFI > 3)	0.305	(0.069, 0.525)
Hospital Frailty Risk Score (HFRS> 5)	Fried Index (FI)	0.025	(−0.132, 0.195)
	Trauma-Specific Frailty Index (TSFI)	0.204	(0.005, 0.398)
	11-Item Modified Frailty Index (mFI)	0.186	(−0.003, 0.373)

## Discussion

Our study’s results show that the agreement of frailty ratings between our GTFI and other frailty indexes ranged from fair to moderate. This level of consistency with frailty scales is not uncommon, as was previously shown when the ICC and kappa consistency were compared with the Fried index, mFI, and TSFI, which ranged from 0.256 to 0.407, and 0.188 to 0.460, respectively, depending on the measurement approach [14]. This range of scores highlights the challenges of using any frailty scale to diagnose an individual as frail [8]. However, the ICC and kappa consistency between the GTFI and HFRS were 0.716 and 0.608, respectively, which was higher than the agreement with the other three indexes. This is likely because the GTFI is constructed based on the patient’s ICD-10 diagnostic code, which is consistent with HFRS, and the construction principles are different from the Fried frailty index based on Fried frailty phenotype, mFI, and TSFI based on multi-dimensional health status [8]. This is consistent with the results of previous research [14].

Aguayo et al. conducted a study on the consistency of the 35 frailty indexes in the British Longitudinal Aging Study in 2016 and showed that the consistency of the various frailty indexes was quite different (Cohen’s kappa value 0.10–0.83). [14] The study suggested that the various frailty indexes have obvious heterogeneity in assessing and identifying the frailty of specific individuals, which supports the findings of our study [14]. This is likely because different frailty indexes are developed based on different concepts of frailty, and most frailty indexes are different from each other. Thus, they cannot be used interchangeably to each other, which leads to poor consistency between frailty indexes constructed based on different concepts [14].

Studies have concluded that there is still no gold standard for assessing frailty [14]. This suggests that it is still challenging to construct a frailty assessment method. However, compared with other frailty evaluation scales, our GTFI has the advantage as it can use routine data from hospital electronic medical record systems and can eliminate the need to manually calculate the score. Although indexes such as the Fried index and TSFI can be convenient and fast to calculate [15], they still require manual data collection and evaluation, which has potential application burdens.

Its weak consistency with the existing frailty indexes does not mean that the GTFI is not effective in judging patients’ frailty. Further studies should focus on the practical application of the GTFI on clinical treatment in hospitals, which is a limitation of this study.

## Conclusions

Different frailty scores are based on different concepts of frailty, and most cannot be assumed to be interchangeable. There is still no gold standard for the current assessment methods of frailty, but it can be compared based on the understanding in terms of the concepts and measures used in each.

## Supplementary Information

**Additional file 1: Appendix 1**. Variables used to construct Fried Index. **Appendix 2**. Variables used to construct Trauma-Specific Frailty Index. **Appendix 3**. Variables used to construct 11-item modified frailty index^a^. **Appendix 4**. List of ICD-10 codes of Hospital Frailty Risk Score. **Appendix 5**. List of ICD-10 codes of Geriatric Trauma Frailty Index.

## Data Availability

No additional data are available.

## Abbreviations

GTFI: Geriatric trauma frailty index
HFRS: Hospital frailty risk score
ICC: Intraclass correlation coefficient
ICD-10: International Statistical Classification of Disease and Related Health Problems 10th Revision
LOS: Length of stay
mFI: Modified frailty index
TSFI: Trauma-specific frailty index
NEDS: National Emergency Department Sample
STEMA: Shanghai Trauma Emergency Medical Association
