# Supplementary material for: Validating the agreement between the geriatric trauma frailty index and four published frailty scores in the Chinese geriatric trauma population
Source: BMC Geriatr. 2022 Mar 4;22:183. doi: 10.1186/s12877-022-02819-9 (PMC8897853; doi:10.1186/s12877-022-02819-9)
Supplement: Supplementary file 1 — Additional file 1: Appendix 1. Variables used to construct Fried Index. Appendix 2. Variables used to construct Trauma-Specific Frailty Index. Appendix 3. Variables used to construct 11-item modified frailty indexa. Appendix 4. List of ICD-10 codes of Hospital Frailty Risk Score. Appendix 5. List of ICD-10 codes of Geriatric Trauma Frailty Index. [file 12877_2022_2819_MOESM1_ESM.docx]

*Appendix 1 Variables used to construct Fried Index*

| **Item** | **Details** | **Rules** |
| --- | --- | --- |
| Nutritional status | Weight loss > 5kg in preceding year | "Yes" - weight_loss_gt5_12mths = 1 |
| Strength | Grip strength, lowest 20% in this population | Max grip strength value from left and right grip strength values. Lowest 20% of values from population stratified by gender and BMI = 1. Male BMI <=24, 24.1-26, 26.1-28, 28+. Female BMI <=23, 23.1-26, 26.1-29, 29+. |
| Energy | Do you feel full of energy? "no" | "No" – energy |
| Mobility | Gait speed 2.4m walk, lowest 20% | Slowest 20% of values stratified by gender and height = 1. Male Height <=173,>173. Female Height <=159,>159. If not able to walk 2.4m then given value of 1. |
| Physical activity | EuroQol-5D questions mobility, self- care, usual activities | Sum EQ5D values for self-care, mobility and usual activities. Score >=7/9 = 1. |

*Appendix 2 Variables used to construct Trauma-Specific Frailty Index*

We changed “Sexual activie” to “Stooping/crouching problem” in Function, and “Albumin” to “Lost weight > 10 pounds in last year” in Nutrition, regarding to the.

| **Item** | **Criterions** | | |
| --- | --- | --- | --- |
| **Comorbidities** |  |  |  |
| Cancer History | Yes (1) | No (0) |  |
| Coronary Heart Disease | MI (1) | CABG (0.75) | PCI (0.5) |
|  | Medication (0.25) | None (0) |  |
| Dementia | Severe (1) | Moderate (0.5) | Mild (0.25) |
|  | No (0) |  |  |
| **Daily Activities** |  |  |  |
| Help with grooming | Yes (1) | No (0) |  |
| Help managing money | Yes (1) | No (0) |  |
| Help doing household work | Yes (1) | No (0) |  |
| Help toileting | Yes (1) | No (0) |  |
| Help walking | Wheelchair (1) | Walker (0.75) | Cane (0.25) |
|  | No (0) |  |  |
| **Health Attitude** |  |  |  |
| Feel less useful | Most time (1) | Sometimes (0.5) | Never (0) |
| Feel sad | Most time (1) | Sometimes (0.5) | Never (0) |
| Feel effort to do everything | Most time (1) | Sometimes (0.5) | Never (0) |
| Falls | Most time (1) | Sometimes (0.5) | Never (0) |
| Feel lonely | Most time (1) | Sometimes (0.5) | Never (0) |
| **Function** |  |  |  |
| Stooping/crouching problem | Yes (1) | No (0) |  |
| **Nutrition** |  |  |  |
| Lost weight > 10 pounds in last year | Yes (1) | No (0) |  |

*Appendix3 Variables used to construct 11-item modified frailty index^a^*

| **NO.** | **Variable** |
| --- | --- |
| 1 | Diabetes mellitus — insulin and non-insulin dependent |
| 2 | Congestive heart failure |
| 3 | Hypertension requiring medication |
| 4 | History of myocardial infarction |
| 5 | Previous percutaneous coronary intervention or angina |
| 6 | History of transient ischemic attack or cerebrovascular accident without neurological deficit |
| 7 | Cerebrovascular accident with neurological deficit |
| 8 | Impaired sensorium |
| 9 | History of chronic obstructive pulmonary disease or pneumonia |
| 10 | History peripheral vascular disease or rest pain |
| 11 | Function health status before surgery — partially or totally dependent for activities of daily living |

a Scores are calculated by adding 1 point for each variable present and then dividing this number by 11.

*Appendix 4 List of ICD-10 codes of Hospital Frailty Risk Score*

| **ICD Code** | **ICD Description** | **Points awarded** |
| --- | --- | --- |
| **F00** | Dementia in Alzheimer's disease | 7.1 |
| **G81** | Hemiplegia | 4.4 |
| **G30** | Alzheimer's disease | 4.0 |
| **I69** | Sequelae of cerebrovascular disease (secondary codes) | 3.7 |
| **R29** | Other symptoms and signs involving the nervous and musculoskeletal systems (R29·6 Tendency to fall) | 3.6 |
| **N39** | Other disorders of urinary system (includes urinary tract infection and urinary incontinence) | 3.2 |
| **F05** | Delirium, not induced by alcohol and other psychoactive substances | 3.2 |
| **W19** | Unspecified fall | 3.2 |
| **S00** | Superficial injury of head | 3.2 |
| **R31** | Unspecified haematuria | 3.0 |
| **B96** | Other bacterial agents as the cause of diseases classified to other chapters (secondary code) | 2.9 |
| **R41** | Other symptoms and signs involving cognitive functions and awareness | 2.7 |
| **R26** | Abnormalities of gait and mobility | 2.6 |
| **I67** | Other cerebrovascular diseases | 2.6 |
| **R56** | Convulsions, not elsewhere classified | 2.6 |
| **R40** | Somnolence, stupor and coma | 2.5 |
| **T83** | Complications of genitourinary prosthetic devices, implants and grafts | 2.4 |
| **S06** | Intracranial injury | 2.4 |
| **S42** | Fracture of shoulder and upper arm | 2.3 |
| **E87** | Other disorders of fluid, electrolyte and acid base balance | 2.3 |
| **M25** | Other joint disorders, not elsewhere classified | 2.3 |
| **E86** | Volume depletion | 2.3 |
| **R54** | Senility | 2.2 |
| **Z50** | Care involving use of rehabilitation procedures | 2.1 |
| **F03** | Unspecified dementia | 2.1 |
| **W18** | Other fall on same level | 2.1 |
| **Z75** | Problems related to medical facilities and other health care | 2.0 |
| **F01** | Vascular dementia | 2.0 |
| **S80** | Superficial injury of lower leg | 2.0 |
| **L03** | Cellulitis | 2.0 |
| **H54** | Blindness and low vision | 1.9 |
| **E53** | Deficiency of other B group vitamins | 1.9 |
| **Z60** | Problems related to social environment | 1.8 |
| **G20** | Parkinson's disease | 1.8 |
| **R55** | Syncope and collapse | 1.8 |
| **S22** | Fracture of rib(s), sternum and thoracic spine | 1.8 |
| **K59** | Other functional intestinal disorders | 1.8 |
| **N17** | Acute renal failure | 1.8 |
| **L89** | Decubitus ulcer | 1.7 |
| **Z22** | Carrier of infectious disease | 1.7 |
| **B95** | Streptococcus and staphylococcus as the cause of diseases classified to other chapters | 1.7 |
| **L97** | Ulcer of lower limb, not elsewhere classified | 1.6 |
| **R44** | Other symptoms and signs involving general sensations and perceptions | 1.6 |
| **K26** | Duodenal ulcer | 1.6 |
| **I95** | Hypotension | 1.6 |
| **N19** | Unspecified renal failure | 1.6 |
| **A41** | Other septicaemia | 1.6 |
| **Z87** | Personal history of other diseases and conditions | 1.5 |
| **J96** | Respiratory failure, not elsewhere classified | 1.5 |
| **X59** | Exposure to unspecified factor | 1.5 |
| **M19** | Other arthrosis | 1.5 |
| **G40** | Epilepsy | 1.5 |
| **M81** | Osteoporosis without pathological fracture | 1.4 |
| **S72** | Fracture of femur | 1.4 |
| **S32** | Fracture of lumbar spine and pelvis | 1.4 |
| **E16** | Other disorders of pancreatic internal secretion | 1.4 |
| **R94** | Abnormal results of function studies | 1.4 |
| **N18** | Chronic renal failure | 1.4 |
| **R33** | Retention of urine | 1.3 |
| **R69** | Unknown and unspecified causes of morbidity | 1.3 |
| **N28** | Other disorders of kidney and ureter, not elsewhere classified | 1.3 |
| **R32** | Unspecified urinary incontinence | 1.2 |
| **G31** | Other degenerative diseases of nervous system, not elsewhere classified | 1.2 |
| **Y95** | Nosocomial condition | 1.2 |
| **S09** | Other and unspecified injuries of head | 1.2 |
| **R45** | Symptoms and signs involving emotional state | 1.2 |
| **G45** | Transient cerebral ischaemic attacks and related syndromes | 1.2 |
| **Z74** | Problems related to care-provider dependency | 1.1 |
| **M79** | Other soft tissue disorders, not elsewhere classified | 1.1 |
| **W06** | Fall involving bed | 1.1 |
| **S01** | Open wound of head | 1.1 |
| **A04** | Other bacterial intestinal infections | 1.1 |
| **A09** | Diarrhoea and gastroenteritis of presumed infectious origin | 1.1 |
| **J18** | Pneumonia, organism unspecified | 1.1 |
| **J69** | Pneumonitis due to solids and liquids | 1.0 |
| **R47** | Speech disturbances, not elsewhere classified | 1.0 |
| **E55** | Vitamin D deficiency | 1.0 |
| **Z93** | Artificial opening status | 1.0 |
| **R02** | Gangrene, not elsewhere classified | 1.0 |
| **R63** | Symptoms and signs concerning food and fluid intake | 0.9 |
| **H91** | Other hearing loss | 0.9 |
| **W10** | Fall on and from stairs and steps | 0.9 |
| **W01** | Fall on same level from slipping, tripping and stumbling | 0.9 |
| **E05** | Thyrotoxicosis [hyperthyroidism] | 0.9 |
| **M41** | Scoliosis | 0.9 |
| **R13** | Dysphagia | 0.8 |
| **Z99** | Dependence on enabling machines and devices | 0.8 |
| **U80** | Agent resistant to penicillin and related antibiotics | 0.8 |
| **M80** | Osteoporosis with pathological fracture | 0.8 |
| **K92** | Other diseases of digestive system | 0.8 |
| **I63** | Cerebral Infarction | 0.8 |
| **N20** | Calculus of kidney and ureter | 0.7 |
| **F10** | Mental and behavioural disorders due to use of alcohol | 0.7 |
| **Y84** | Other medical procedures as the cause of abnormal reaction of the patient | 0.7 |
| **R00** | Abnormalities of heart beat | 0.7 |
| **J22** | Unspecified acute lower respiratory infection | 0.7 |
| **Z73** | Problems related to life-management difficulty | 0.6 |
| **R79** | Other abnormal findings of blood chemistry | 0.6 |
| **Z91** | Personal history of risk-factors, not elsewhere classified | 0.5 |
| **S51** | Open wound of forearm | 0.5 |
| **F32** | Depressive episode | 0.5 |
| **M48** | Spinal stenosis (secondary code only) | 0.5 |
| **E83** | Disorders of mineral metabolism | 0.4 |
| **M15** | Polyarthrosis | 0.4 |
| **D64** | Other anaemias | 0.4 |
| **L08** | Other local infections of skin and subcutaneous tissue | 0.4 |
| **R11** | Nausea and vomiting | 0.3 |
| **K52** | Other non-infective gastroenteritis and colitis | 0.3 |
| **R50** | Fever of unknown origin | 0.1 |

*Appendix 5 List of ICD-10 codes of Geriatric Trauma Frailty Index*

| **ICD Code** | **ICD Description** | **Points awarded** |
| --- | --- | --- |
| A04 | Other bacterial intestinal infections | 0.937 |
| A41 | Other septicemia | 0.682 |
| B37 | candidiasis | 0.83 |
| D68 | Other coagulation defects | 0.445 |
| E43 | Unspecified severe protein-energy malnutrition | 0.714 |
| E44 | Protein-energy malnutrition of moderate and mild degree | 0.773 |
| E46 | Unspecified protein-energy malnutrition | 1.994 |
| E87 | Other disorders of fluid, electrolyte and acid-base balance | 0.602 |
| F05 | Delirium, not induced by alcohol and other psychoactive substances | 1.188 |
| G81 | Hemiplegia | 0.9 |
| G92 | Toxic encephalopathy | 0.579 |
| G93 | Other disorders of brain | 0.572 |
| I21 | Acute myocardial infarction | 0.33 |
| I26 | Pulmonary embolism | 0.978 |
| I46 | Cardiac arrest | 0.067 |
| I47 | Paroxysmal tachycardia | 0.883 |
| I63 | Cerebral infarction | 0.61 |
| I82 | Other venous embolism and thrombosis | 1.04 |
| J15 | Bacterial pneumonia, not elsewhere classified | 0.952 |
| J18 | Pneumonia, organism unspecified | 0.859 |
| J69 | Pneumonitis due to solids and liquids | 0.642 |
| J90 | Pleural diffusion, not elsewhere classified | 0.433 |
| J95 | Postprocedural respiratory disorders, not elsewhere classified | 0.792 |
| J96 | Respiratory failure, not elsewhere classified | 0.893 |
| J98 | Other respiratory disorders | 0.277 |
| K56 | Paralytic ileus and intestinal obstruction without hernia | 1.033 |
| K92 | Other disease of digestive system | 1.027 |
| L89 | Decubitus ulcer and pressure area | 0.319 |
| N17 | Acute renal failure | 0.418 |
| R13 | Dysphagia | 0.999 |
| R40 | Somnolence, stupor and coma | 1.333 |
| R47 | Speech disturbances, not elsewhere classified | 0.723 |
| R56 | Convulsions, not elsewhere classified | 0.536 |
| R57 | Shock, not elsewhere classified | 0.368 |
| R64 | Cachexia | 0.054 |
| R65 | Systemic inflammatory response syndrome | 0.3 |
| S06 | Intracranial injury | 0.537 |
| S14 | Injury of nerves and spinal cord at neck level | 0.961 |
| T79 | Certain early complications of trauma, not elsewhere classified | 0.221 |
| T84 | Complications of internal orthopedic prosthetic devices, implants and grafts | 1.304 |
| Z51 | Other medical care | 0.493 |
| Z81 | Family history of mental and behavioral disorders | 0.708 |
